# Sialic acid biosensing by post-printing modification of PEDOT:PSS with pyridylboronic acid

**DOI:** 10.1080/14686996.2022.2122867

**Published:** 2022-09-16

**Authors:** Hideki Fujisaki, Akira Matsumoto, Yuji Miyahara, Tatsuro Goda

**Affiliations:** a Institute of Biomaterials and Bioengineering, Tokyo Medical and Dental University, Tokyo, Japan; b Department of Materials Engineering, Graduate School of Engineering, The University of Tokyo, Tokyo, Japan; c Department of Research and Development, Kanagawa Institute of Industrial Science and Technology (KISTEC), Tokyo, Japan; d Department of Biomedical Engineering, Faculty of Science and Engineering, Toyo University, Saitama, Japan

**Keywords:** PEDOT:PSS, potentiometry, surface modification, sialic acid, pyridylboronic acid

## Abstract

A poly(3,4-ethylenedioxythiophene):poly(styrenesulfonate) (PEDOT:PSS)-based conducting polymer, which has biorecognition capabilities, has promising biosensing applications. Previously, we developed a facile method for post-printing chemical modification of PEDOT:PSS thin films from commercial sources. Molecular recognition elements were directly introduced into the PSS side chain by a two-step chemical reaction: introduction of an ethylenediamine linker via an acid chloride reaction of the sulfonate moiety, and subsequent receptor attachment to the linker via amine coupling. In this study, the same method was used to introduce 6-carboxypyridine-3-boronic acid (carboxy-PyBA) into the linker for specifically detecting *N*-acetylneuraminic acid (sialic acid, SA), as a cancer biomarker. The surface-modified PEDOT:PSS films were characterized by X-ray photoelectron spectroscopy, attenuated total reflection Fourier-transform infrared spectroscopy, and static water contact angle and conductivity measurements. The specific interaction between PyBA and SA was detected by label-free reagent-free potentiometry. The SA-specific negative potential responses of modified PEDOT:PSS electrodes, which was ascribed to an SA carboxyl anion, were observed in a physiologically relevant SA range (1.6–2.9 mM) at pH 5, in a concentration-dependent manner even in the presence of 10% fetal bovine serum. The sensitivity was −2.9 mV/mM in 1–5 mM SA with a limit of detection of 0.7 mM. The sensing performances were almost equivalent to those of existing graphene-based electrical SA sensors. These results show that our chemical derivatization method for printing PEDOT:PSS thin films will have applications in SA clinical diagnostics.

## GRAPHICAL ABSTRACT

**Figure:**
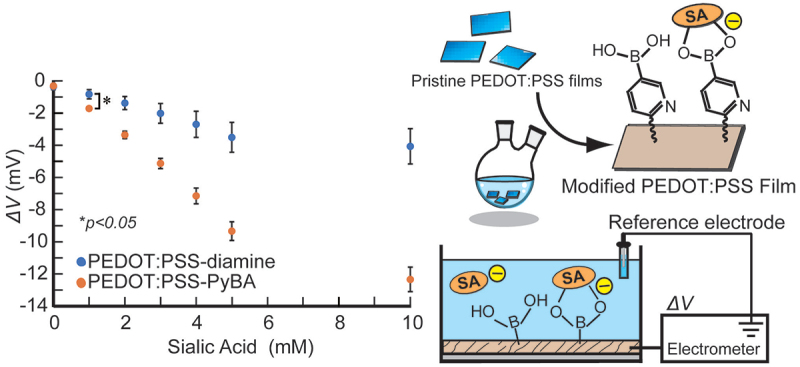

## 1. Introduction

Poly(3,4-ethylenedioxythiophene):poly(styrenesulfonate) (PEDOT:PSS) is a thin, flexible, printable material with high conductivity, robustness, processability, and biocompatibility [1–6]. These advantages enable its use in various applications in biomedical engineering, such as wearable sensing [7–25]. Medical sensors are mainly classified into three types based on their measurement type: physical, chemical, and biosensors. Physical sensors include electrocardiographs, electromyographs, and electroencephalographs [26–28]. Chemical sensors detect volatile organic compounds, biogases, physiological ions, and metabolites [29–31]. A limited number of biosensor applications that use PEDOT:PSS have been reported. This is because of the unique requirements of biosensing materials. Target specificity is necessary for biosensing, and therefore biosensors consist of a biorecognition layer and a signal transducer. The biorecognition layer includes target recognition elements such as antibodies, enzymes, and aptamers. However, PEDOT:PSS lacks the ability to achieve specific biorecognition, therefore it is unsuitable for biosensing applications in its original form. Facile methods for the introduction of biorecognition elements into PEDOT:PSS will therefore enable its use in a wide range of biosensing applications.

There are two main methods for endowing PEDOT:PSS with biorecognition capabilities. The first is to blend it with a substance that has biorecognition abilities. For example, glucose oxidase has been blended with PEDOT:PSS for specific glucose biosensing [32]. The advantages of this method are its simplicity and cost efficiency. Blending occasionally enhances conductivity [32,33]. However, without fixation, bioreceptors are subject to being released from the matrix. The biorecognition of a protein is easily lost by denaturation during fabrication and long-term usage [32]. The second method is to introduce a biorecognition site into the side chain of PEDOT or PSS via chemical synthesis of PEDOT:PSS derivatives. Hydroxymethyl-PEDOT (PEDOT-OH) enables covalent immobilization of anti-*Escherichia coli* by using an epoxysilane [34]. Similarly, the covalent immobilization of glucose oxidase has been achieved by epoxysilane treatment to give a poly(vinyl alcohol)/PEDOT:PSS blend [35]. Our group synthesized a PEDOT derivative that contains a phospholipid polar group in the side chain for detecting the inflammation biomarker C-reactive protein [36]. In addition, α2,6-sialyllactose was conjugated with a PEDOT derivative bearing an oxyamine moiety for specific recognition of human influenza A virus [37]. In this way, various PEDOT analogs can be synthesized for biosensing applications. The conductivities of PEDOT adducts are lower than that of pristine PEDOT [38–40].

We have been investigating new methods for obtaining PEDOT:PSS derivatives that possess bioreceptors. In particular, post-printing chemical modification is attractive in terms of simplicity and cost efficiency. Post-printing modification of PSS by using aromatic diazonium salts has been reported [41], but the complexity of the chemical reaction is an issue. Previously, as a proof of concept, we performed a two-step post-printing modification of PSS [42]. First, an ethylenediamine linker was introduced into the sulfonate group via an acid chloride reaction. Then, 4-carboxy-3-fluorophenylboronic acid (carboxy-FPBA) was introduced into the amine end by an amide condensation reaction. FPBA functioned as a synthetic receptor for diol compounds and enabled label-free glucose sensing. Our post-printing modification has the advantages of simplicity, mass productivity, and cost efficiency. Furthermore, by changing the linker and biorecognition element, it can be used to detect a wide variety of analytes. In this study, to demonstrate the versatility of our developed technique, we introduced a pyridylboronic acid (PyBA) bioreceptor into printed PEDOT:PSS films for specifically detecting *N*-acetylneuraminic acid, namely sialic acid (SA). SA is recognized as inflammation [43–45] and cancer metastasis [46–49] biomarkers. In recent years, SA has been reported to be related to brain diseases [50,51]. The artificial bioreceptor PyBA specifically interacts with SA under weakly acidic conditions among other monosaccharides including glucose [52]. This interaction enables specific SA detection by using PyBA-functionalized PEDOT:PSS (PEDOT:PSS-PyBA). Compared with natural lectins, synthetic PyBA is stable and robust, and is suitable for biosensing in many biomedical fields.

## 2. Materials and methods

### 2.1. Materials

A poly(ethylene terephthalate) (PET) substrate-laminated PEDOT:PSS (200 nm thick) thin film (Orgacon® F350, Agfa-Gebhardt, Tokyo, Japan) was used. Oxalyl chloride was purchased from Tokyo Chemical Industry (Tokyo, Japan). 6-Carboxypyridine-3-boronic acid, 4-(4,6-dimethoxy-1,3,5-triazin-2-yl)-4-methylmorpholinium chloride (DMT-MM), and SA were purchased from Fujifilm Wako Pure Chemicals (Tokyo, Japan). Fetal bovine serum (FBS) was purchased from Funakoshi (Tokyo, Japan). All other reagents were of extra-pure grade, obtained from commercial sources, and used as received. Milli-Q water (18.2 MΩ cm, Merck, Darmstadt, Germany) was used in all experiments.

### 2.2. Chemical modification of PEDOT:PSS film

**Figure 1.**
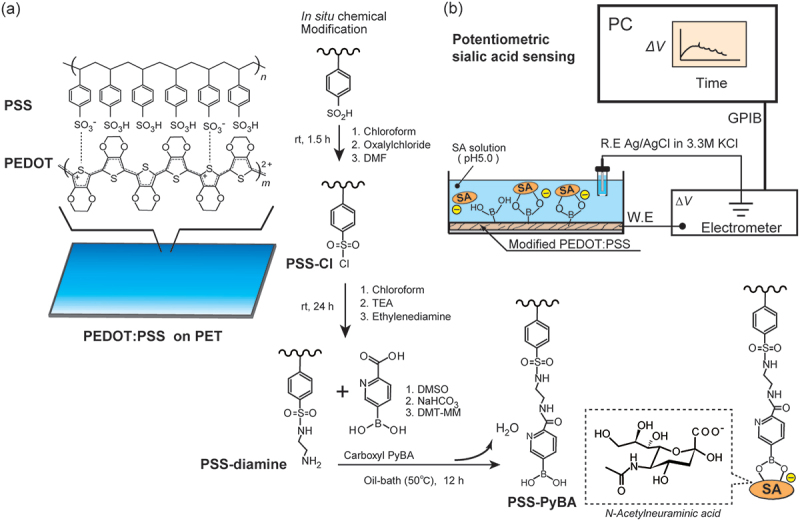
*In situ* post-printing chemical derivatization of PEDOT:PSS layer on PET substrate for label-free biosensing of SA. The sulfonate group in PEDOT:PSS was converted into a sulfonate chloride, with subsequent introduction of carboxy-PyBA via ethylenediamine as a linker. SA was recognized by PyBA-modified PEDOT:PSS. The specific binding of anionic SA generated negative surface potentials in potentiometric measurements.

The PEDOT:PSS film on PET was modified via a two-step reaction (Figure 1(a)) [53]. The first step was an acid chloride reaction at the sulfonate group in PSS, with oxalyl chloride, and subsequent introduction of ethylenediamine, as previously reported [42]. In the second step, dimethyl sulfoxide (10 mL), sodium hydrogen carbonate (42 mg), and 6-carboxypyridine-3-boronic acid (184 mg, 1.1 mmol) were added to a two-necked flask (50 mL). The ethylenediamine-modified PEDOT:PSS (PEDOT:PSS-diamine) and DMT-MM (304 mg, 1.1 mmol) were placed in the two-necked flask under Ar, and the reaction was performed for 12 h at 50°C. The films were washed with water and dried for 1 h at 25°C. The 6-carboxypyridine-3-boronic acid-modified PEDOT:PSS-diamine film (PEDOT:PSS-PyBA) was stored in the dark at 25°C until use.

### 2.3. Characterization

The film surfaces were examined by X-ray photoelectron spectroscopy (XPS; JPS-9010MC spectrometer, JEOL Ltd., Tokyo, Japan). An Mg Kα source (12 kV) and a neutralizer were used. The photoelectron pass energy was 20 eV and the takeoff angle was 90°. Charge correction was based on the C1s peak at 284.2 eV. The mean and standard deviation (SD) were obtained from three measurements.

The surface wettability was evaluated by performing static water contact angle measurements (DM-501 goniometer, Kyowa Interfacial Chemical, Saitama, Japan). The mean and SD were calculated from five measurements.

The surface resistance was measured by the two-terminal method at a 1 mm distance by using a 107 digital multimeter (FLUKE, Everett, WA, USA) with 0.7 mm tip, gold-coated low-resistance probes. The mean and SD were obtained from ten measurements.

An iS50 Fourier-transform infrared (FTIR) spectrometer (Thermo Fisher Scientific Japan, Tokyo, Japan) equipped with a universal attenuated total reflection (ATR) sampling assembly was used to identify the functional groups on the surfaces of the modified PEDOT:PSS films.

### 2.4. Potentiometry

The modified PEDOT:PSS films (1 cm × 1 cm), which were used as working electrodes, and an Ag/AgCl reference electrode (in 3.3 M KCl_aq_ via a salt bridge) were connected to a 6517B multichannel high-resistance electrometer (Keithley Instruments, Cleveland, OH, USA). The working and reference electrodes were immersed in a 50 mM 2-(*N*-morpholino)ethanesulfonic acid (MES) buffer solution (pH 5.0) (Figure 1(b)). After conditioning for 2 h, a stock solution of SA was added dropwise at 20 min intervals to increase the SA concentration from 0 to 1, 2, 3, 4, 5, and 10 mM, while potential changes (*ΔV*) from baseline (stable points just before treating the solution at 0 mM SA) were observed. The interval was set for 20 min at each SA level to minimize the effect of signaling drift over time while assuring the period for the binding equilibrium. The same method was used to examine the potentiometric responses to glucose, galactose, and mannose. The same potentiometric system was used to investigate the pH dependences in buffer solutions at pH 5.0, 6.0, 7.0, and 8.0. MES buffer solutions were used at pH 5.0 and 6.0, and 4-(2-hydroxyethyl)-1-piperazineethanesulfonic acid (HEPES) buffer solutions (10 mM) were used at pH 7.0 and 8.0. A MES buffer containing 10% FBS was used for measurements under realistic conditions. The mean and SD were determined from five measurements.

### 2.5. Statistical analysis

The data were analyzed by using Student’s *t*-tests in two groups. In three or more groups, the data were analyzed by analysis of variance (one-way), and multiple comparisons were made by using Tukey’s test; *p* < 0.05 was considered to be statistically significant.

## 3. Results and discussion

### 3.1. Confirmation of surface modification of PEDOT:PSS films

**Figure 2.**
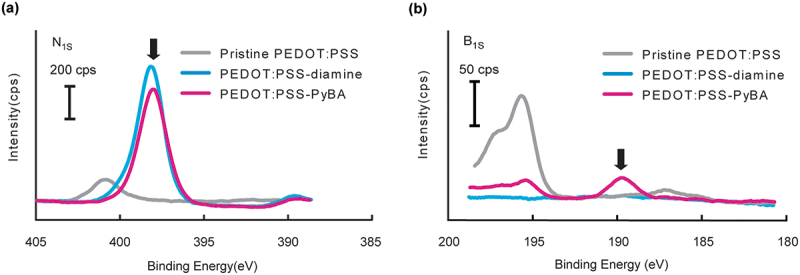
High-resolution XPS data of pristine PEDOT:PSS, PEDOT:PSS-diamine, and PEDOT:PSS-PyBA. (a) N1s peaks in PEDOT:PSS-diamine and PEDOT:PSS-PyBA spectra indicate ethylenediamine or pyridine moieties. (b) distinct B1s peak in PEDOT:PSS-PyBA spectrum indicates covalent introduction of PyBA on the film.

Surface modification of the PEDOT:PSS film in each synthetic step was evaluated by using XPS to perform elemental analysis. The N1s peaks at 398 eV for PEDOT:PSS-diamine and PEDOT:PSS-PyBA were clearly stronger than that for pristine PEDOT:PSS (Figure 2(a)). These results indicate that the acid chloride reaction introduced ethylenediamine into the PSS side chain. However, the nitrogen in the pyridine ring was indistinguishable from that in ethylenediamine. Alternatively, PyBA introduction was confirmed by the presence of the B1s peak at 190 eV (Figure 2(b)). Successful chemical modification of PEDOT:PSS-diamine with carboxy-PyBA via amine coupling condensation was therefore confirmed.

ATR-FTIR spectroscopic analysis also confirmed two-step surface modification (Figure S1). The peaks at 1660 and 3300 cm^−1^, from C(=O)–NH and NH, respectively, indicated the presence of ethylenediamine in PEDOT:PSS-diamine and PEDOT:PSS-PyBA. The peaks at 1700 and 870 cm^−1^, from – C=O and aromatic disubstituents, respectively, were exclusively observed for PEDOT:PSS-PyBA. The peak at 720 cm^−1^, from the benzene ring, was observed for all the samples. The changes in the spectrum of pristine PEDOT:PSS were the same as those previously reported [54,55].

The static water droplet contact angles and surface resistance were measured before and after chemical modifications (Table 1). The contact angles for PEDOT:PSS-diamine and PEDOT:PSS-PyBA were higher than that for pristine PEDOT:PSS. The decreased hydrophilicity indicates that the anionic sulfonate groups were replaced by neutral ethylenediamine and PyBA by chemical modification at pH 7.4. The surface resistances of PEDOT:PSS-diamine and PEDOT:PSS-PyBA were 6.9 and 4.6 times higher, respectively, than that of the pristine PEDOT:PSS film as a result of the chemical modifications. Low-impedance surfaces are important for sensitive electrochemical biosensing. These values are comparable to those previously reported for successful potentiometric biosensing of glucose by using chemically modified PDOT:PSS with a surface resistance of 10^3^ Ω/□ [42]. We confirmed that a film with a surface resistance of the order of 10^4^ Ω/□ gave no potentiometric response.

**Table 1.** Changes in static water contact angle and sheet resistance during surface modification. Water contact angle: mean ± SD (*n* = 5); sheet resistance: mean ± SD (*n* = 10).

Entry	Water Contact Angles (°)	Sheet Resistance (Ω/□)*
Pristine PEDOT:PSS	10.9 ± 0.2	350 ± 4
PEDOT:PSS-diamine	56.2 ± 1.1	2420 ± 610
PEDOT:PSS-PyBA	55.1 ± 0.4	1610 ± 270

*Surface resistance per unit area (1 cm^2^).

The surface morphology of PEDOT:PSS before and after chemical modifications were observed by scanning electron microscopy (SEM). A smooth surface was observed on the pristine PEDOT:PSS. On the other hand, rough surfaces were observed on PEDOT:PSS-diamine and PEDOT:PSS-PyBA (Figure S2). These topographical changes may have influenced on the wettability and sensitivity.

### 3.2. pH-Dependent SA binding to PEDOT:PSS-PyBA

**Figure 3.**
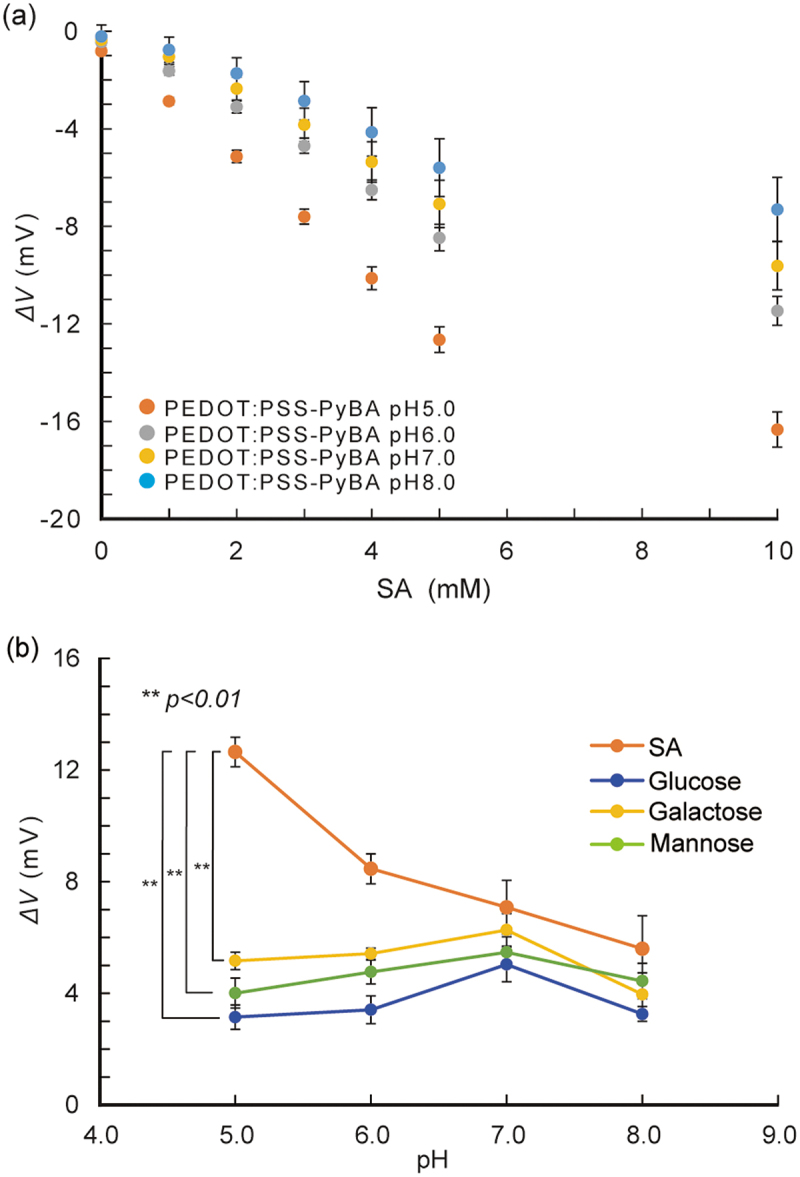
pH-Dependent selective binding of SA with PyBA on PEDOT:PSS-PyBA. (a) potential changes of PEDOT:PSS-PyBA electrodes at varying concentrations of SA from 1 to 10 mM at pH 5.0–8.0 at 25°C; mean ± SD (*n* = 5). (b) potential changes of PEDOT:PSS-PyBA electrodes in 5 mM SA, glucose, galactose, and mannose at pH 5.0–8.0 at 25°C. Mean ± SD (*n* = 5), **p < 0.01.

On the basis of the surface characterization results, we used PEDOT:PSS-PyBA for label-free SA biosensing. Under weakly acidic conditions, the non-dissociated form of PyBA has specificity for SA over other diols, including monosaccharides [56,57]. The pH dependence of SA recognition was investigated by performing potentiometric measurements (Figure 3(a)). The potential changed significantly (*p* < 0.01) in the negative direction with increasing SA concentration at pH 5.0; this trend was not observed at higher pH conditions. The negative potential change indicated binding of negatively charged SA to the non-dissociated form of PyBA in the PEDOT:PSS side chain. The potential changes for 1–10 mM glucose, galactose, and mannose were negligible at pH 5.0 (Figures 3(b) and S3a). These results are consistent with previous reports that these monosaccharides cannot bind to non-dissociated PyBA under weakly acidic conditions, that is, below the p*K*a of PyBA [58–60]. At pH 7.0, the PyBA moiety started to bind to these monosaccharides (Figure S3b). This shows that PEDOT:PSS-PyBA lost its specificity for SA at neutral pH. The negative potential change that followed the binding of electrically neutral monosaccharides is attributed to dissociation of PyBA.

**Figure 4.**
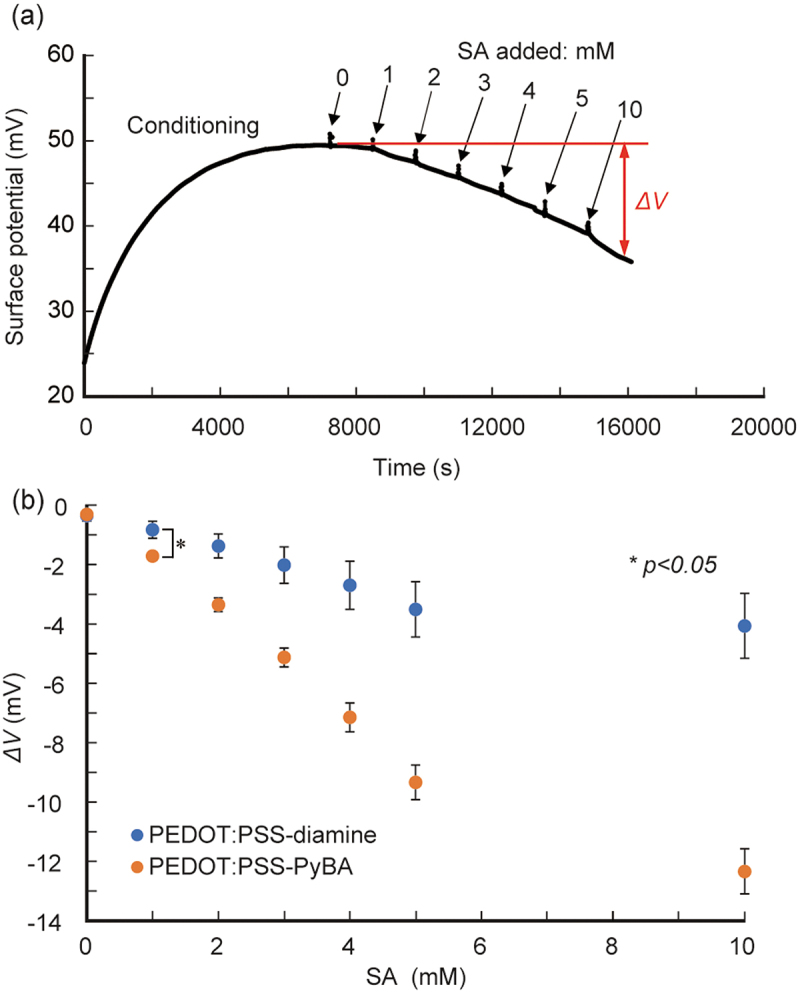
Potentiometric detection of SA using PyBA-functionalized PEDOT:PSS electrodes at pH 5.0 at 25°C. (a) time course of potential changes during sequential increases in SA concentration from 0 to 10 mM. (b) Δ*V* as function of SA concentration for PEDOT:PSS-PyBA and PEDOT:PSS-diamine. Mean ± SD (*n* = 5). Statistically significant differences (*p < 0.05) were observed at ≥1 mM SA.

We then performed real-time label-free SA biosensing. PEDOT:PSS-PyBA rapidly responded to changes in the SA solution concentration, and increasingly negative potential signals were observed (Figure 4(a)). The signal was approximately −12.5 mV at 10 mM SA. In contrast, PEDOT:PSS-diamine, which lacks PyBA as a receptor for SA, gave modest shifts, up to −4 mV at 10 mM SA (Figure S4). These small shifts are considered to be non-specific signals. Statistically significant differences (*p* < 0.05) were observed between PEDOT:PSS-PyBA and PEDOT:PSS-diamine at SA concentration ≥1 mM (Figure 4(b)). PEDOT:PSS-PyBA showed a linear response of −1.9 mV/mM at 1–5 mM SA. The 1:1 Langmuir model was used to analyze the differential signals of the two samples. The apparent dissociation constant (*K*_d_) was 12.1 mM (Figure S5), an order of magnitude inferior to that obtained in the solution phase [60]. The decreased binding ability at the solid/liquid interface can be attributed to steric hindrance and electrostatic repulsion by neighboring SA molecules that already adsorbed on the PyBA receptor. The lower limit of detection of PEDOT:PSS-PyBA was determined to be 0.4 mM, based on three times the standard deviations from the baseline.

### 3.3. SA biosensing in realistic samples

**Figure 5.**
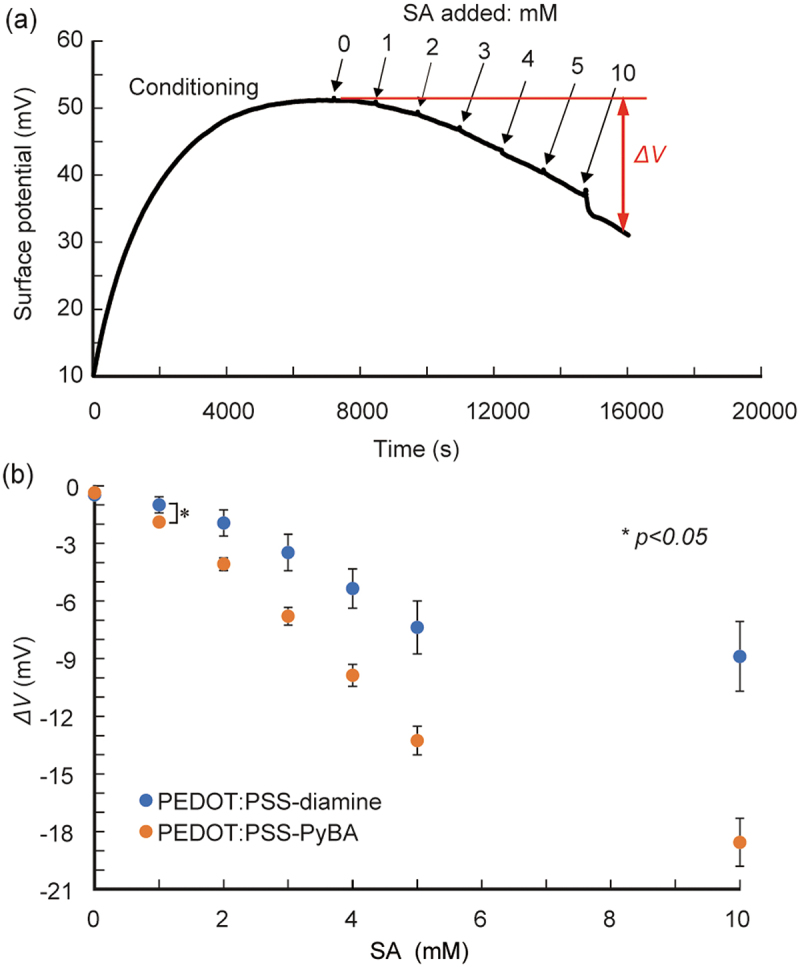
Potentiometric detection of SA using PyBA-functionalized PEDOT:PSS electrodes in 10% FBS at pH 5.0 at 25°C. (a) time course of potential changes during sequential increases in SA concentration. (b) Δ*V* as function of SA concentration for PEDOT:PSS-PyBA and PEDOT:PSS-diamine. Mean ± SD (*n* = 5). Statistically significant differences (*p < 0.05) were observed at ≥1 mM SA.

We performed SA detection in a solution with 10% FBS to confirm the biosensing ability in realistic samples containing interfering substances (e.g. serum proteins) that cause non-specific adsorptions. The same trend was observed; with increasing SA level from 1 to 10 mM in 10% FBS solution, the signals generated by PEDOT:PSS-PyBA were significantly more negative than those generated by PEDOT:PSS-diamine (Figure 5 and S6). The sensitivity of PEDOT:PSS-PyBA was −2.9 mV/mM at 1–5 mM SA. The lower limit of detection was 0.7 mM in 10% FBS. The dynamic range covered clinically relevant free SA levels (1.6–2.9 mM) in human serum [56]. However, with PEDOT:PSS-diamine, the undesired response in 10% FBS was higher than those in buffer solutions. We infer that non-specific adsorption of serum proteins occurred on PEDOT:PSS-diamine, which caused non-specific interactions between the adsorbed layer and SA [61]. It may be necessary to introduce non-fouling molecules such as poly(ethylene glycol) and zwitterions on the surface of the conducting layer [62] to suppress non-specific adsorption of serum proteins.

The sensing performance of PEDOT:PSS-PyBA was compared with those of other potentiometric-type label-free SA biosensors (Table 2). The biosensors listed all cover clinically relevant free SA levels (1.6–2.9 mM) in human serum [56]. The dynamic range of the combined molecular imprinting/carbon nanotube sensor is wider than that of our biosensor, but the sensitivity is comparable. SA biosensors that combine this molecular imprinting technology with carbon nanotubes are expensive, and have a long fabrication time. In contrast, among other biosensors, our biosensor has the advantages of cost efficiency and simplicity, while giving similar sensing performances. These features may enable the development of SA biosensors in wearable formats. Choosing an appropriate combination of a linker and bioreceptor will enable the method described here to be used in a wide range of biosensors with various capture methods.

**Table 2.** Comparison of developed biosensor with potentiometric SA biosensors reported in the literature.

Ligand	Electrode material	Dynamic range (Measurement buffer)	Sensitivity	Applicability	Ref.
PABA	PABA/ERGO/GC	2.0 × 10^−3^ − 1.4 mM (Phosphate buffer)	12 mV/mM		[63]
APBA	APBA/PDABA	0.2–0.91 mM (Phosphate buffer)	—		[61]
3APBA	3APBA – o3ABA	0.03–0.74 mM (Britton – Robinson buffer)	—		[64]
MIP/PABA	SA-imprinted PABA/CNT/GCE	0.08–8.2 mM (Phosphate buffer)	2.2 mV/mM		[65]
MIP/PABA	PABA/CC MIP	0.04–0.44 mM (Phosphate buffer)	0.1 × 10^−3^ mV/mM		[66]
PyBA	PyBA/PEDOT: PSS	0.4–5.0 mM (MES buffer)	1.9 mV/mM	✓	This work

Abbreviations. PABA: poly(aniline boronic acid); ERGO: electrodeposition of reduced graphene oxide; GC: glassy carbon; APBA: aminophenylboronic acid; PDABA: poly(diaminobenzoic acid); 3APBA: 3aminophenylboronic acid; o3ABA: oligomeric 3-aminobenzoic acid; MIP: molecularly imprinted polymer; CNT: carbon nanotube; GCE: glassy carbon electrode; CC: carbon cloth.

## 4. Conclusions

We extended our previously developed method to the chemical modification of PSS side chains with biorecognition sites in a printed PEDOT:PSS layer. PyBA was chemically anchored on the conducting surface, for the first time, via ethylenediamine for specifically detecting SA as a cancer biomarker under clinically relevant conditions. Our results prove the feasibility of introducing a variety of biorecognition elements into large areas of commercial PEDOT:PSS films. These chemically derivatized conducting polymer films have the potential to be commonly used materials in versatile electrochemical biosensing applications. The *in situ* surface modification of flexible PEDOT:PSS thin films could be used to develop wearable devices that could contribute to establishing digital healthcare systems in the future.

## Supplementary Material

Supplemental Material
