# Supplementary material for: Sialic acid biosensing by post-printing modification of PEDOT:PSS with pyridylboronic acid
Source: Sci Technol Adv Mater. 2022 Sep 16;23(1):525–34. doi: 10.1080/14686996.2022.2122867 (PMC9487965; doi:10.1080/14686996.2022.2122867)
Supplement: Supplemental Material [file TSTA_A_2122867_SM8095.docx]

Supplemental Data

**Sialic acid biosensing by post-printing modification of PEDOT:PSS with pyridylboronic acid**

Hideki Fujisaki,^1^ Akira Matsumoto, ^1-3^ Yuji Miyahara,^1^ Tatsuro Goda^*4^

^1^Institute of Biomaterials and Bioengineering, Tokyo Medical and Dental University, 2-3-10 Kanda-Surugadai, Chiyoda, Tokyo 101-0062, Japan

^2^Department of Materials Engineering, Graduate School of Engineering, The University of Tokyo, 7-3-1 Hongo, Bunkyo-ku, Tokyo 113-8656, Japan

^3^Department of Research and Development, Kanagawa Institute of Industrial Science and Technology (KISTEC), 705-1 Shimoimaizumi, Ebina, Kanagawa 243-0435, Japan

^4^Department of Biomedical Engineering, Faculty of Science and Engineering, Toyo University, 2100 Kujirai, Kawagoe, 350-8585 Saitama, Japan

^*^Corresponding Author

Tel: +81-49-239-1746, E-mail: goda@toyo.jp


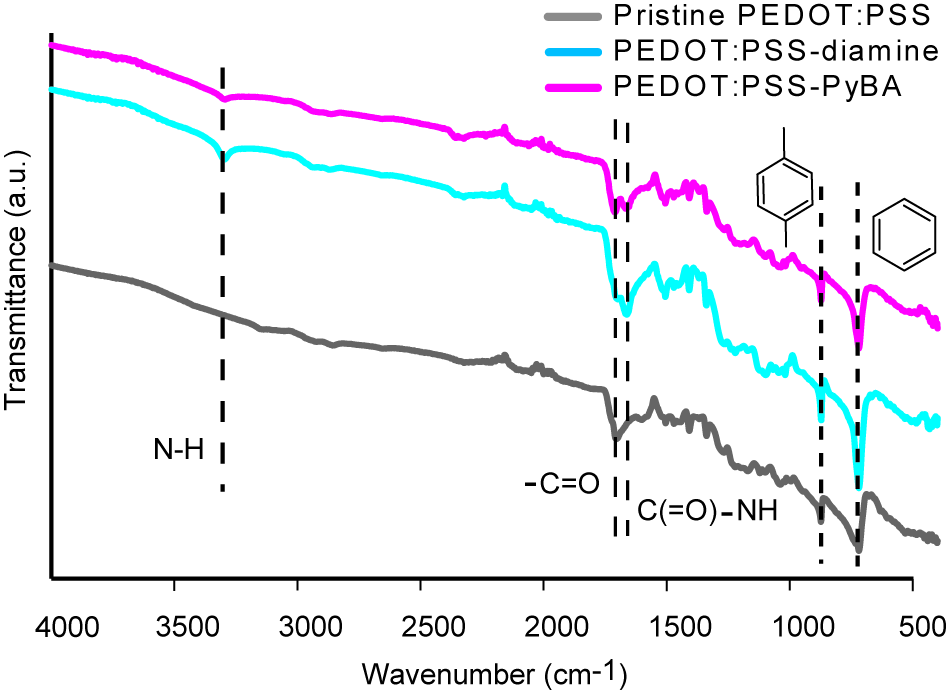


Figure S1. ATR-FTIR spectrum before and after the surface modification of PEDOT:PSS with ethylenediamine (PEDOT:PSS-diamine) and PyBA (PEDOT:PSS-PyBA).

**
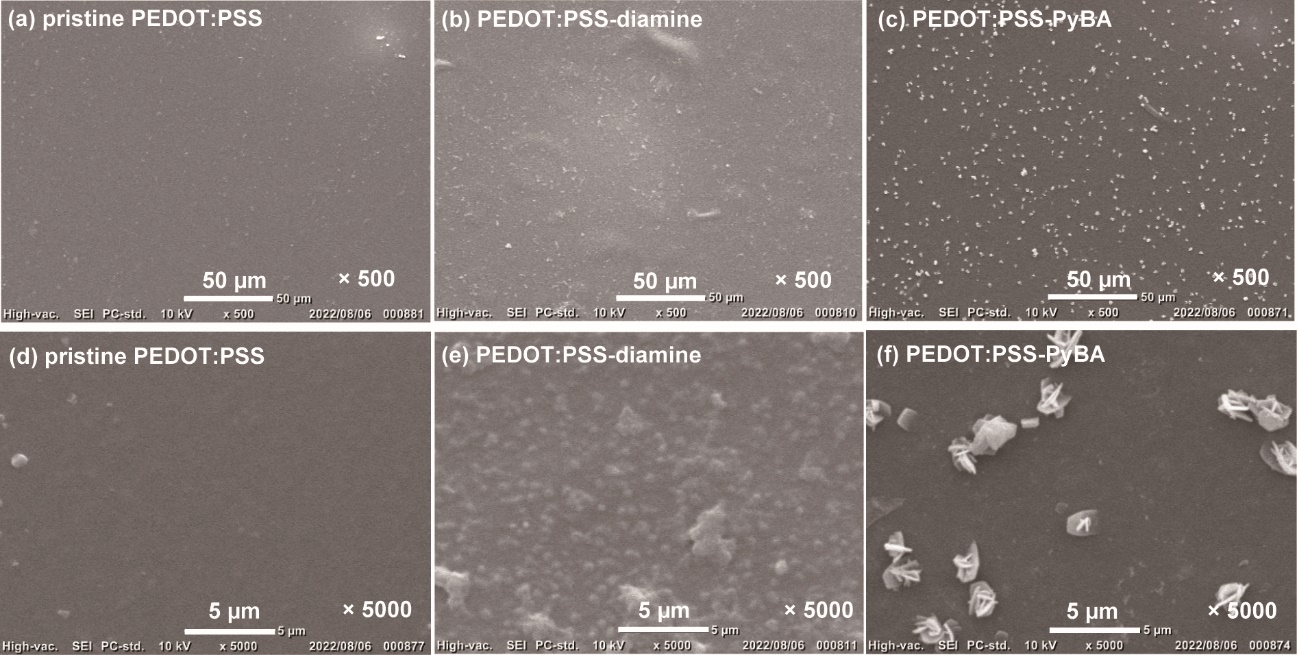
**

Figure S2. Scanning electron microscopy (SEM) images showing surface morphology of the PEDOT:PSS films before and after chemical modifications. (a) Pristine PEDOT:PSS, Magnification: ×500; (b) PEDOT:PSS-diamine, Magnification: ×500; (c) PEDOT:PSS-PyBA, Magnification: ×500; (d) pristine PEDOT:PSS, Magnification: ×5000; (e) PEDOT:PSS-diamine, Magnification: ×5000; (f) PEDOT:PSS-PyBA, Magnification: ×5000.

SEM images were taken by JCM-6000 (JEOL, Tokyo, Japan) with an acceleration voltage of 10 kV, a working distance of 19000 μm (Emission current is automatically set). The samples were treated with a gold coater before the SEM observations.


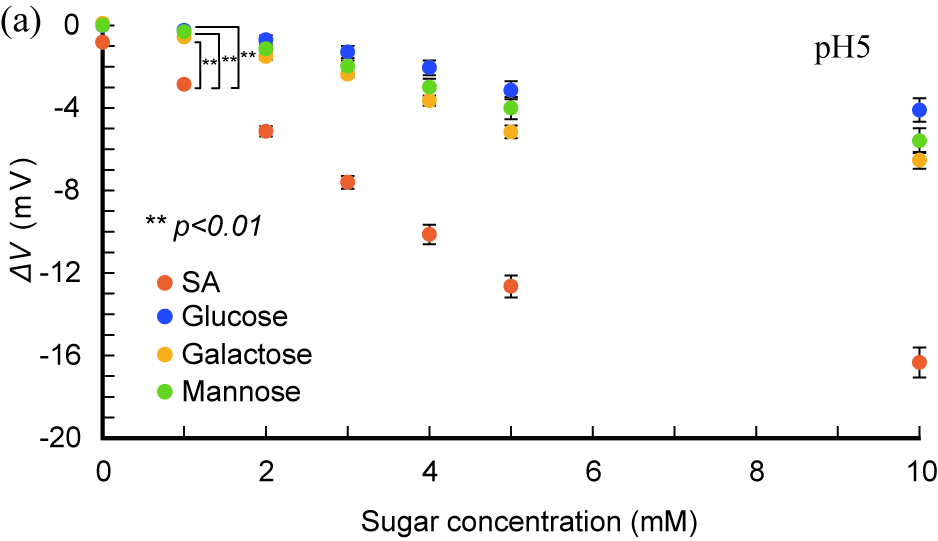


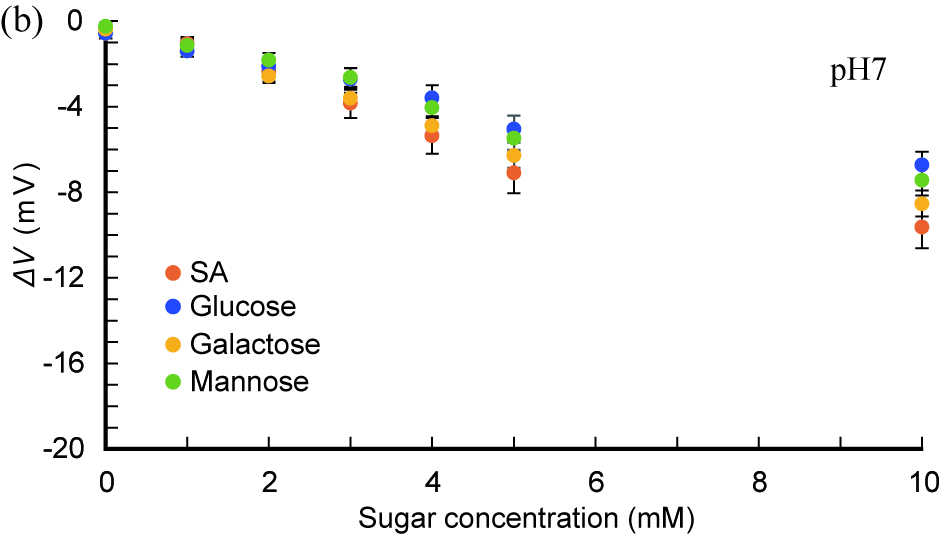


Figure S3. Potential changes (Δ*V*) vs. sugar concentration for PEDOT:PSS-PyBA at pH 5.0 and 7.0 at 25°C. (a) Potential changes from 1 to 10 mM sugar. Mean±SD (*n*=5). Significant difference at ≥ 1 mM, ***p<0.01.* (b) Potential changes from 1 to 10 mM sugar at pH7.0. Mean±SD (*n*=5) .


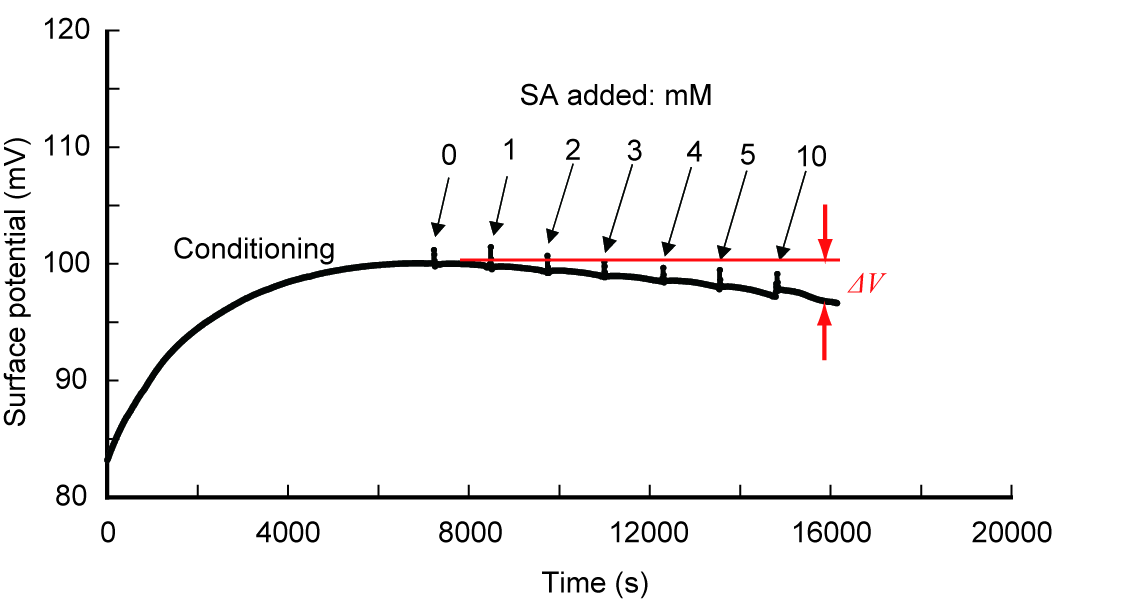


Figure S4. Time-course of potential for PEDOT:PSS-diamine during sequential increase of SA concentration from 0 to 10 mM at 20-min intervals at pH 5.0 at 25̊C. *ΔV* was determined by the difference from the baseline just before treating the solution at 0 mM SA.


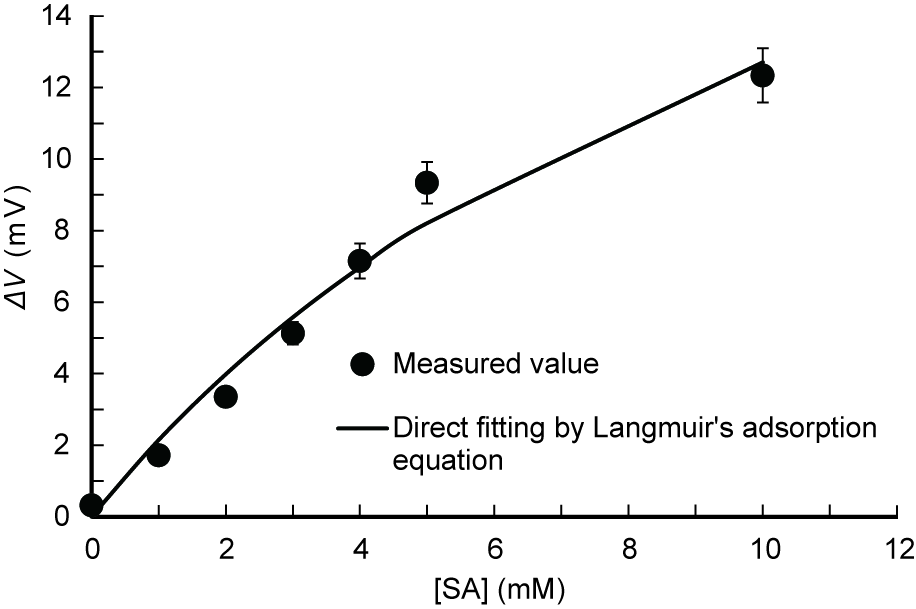


Figure S5. Determination of apparent dissociation constant (*K_d_*) by applying the 1:1 Langmuir adsorption model to potentiometric signal (Δ*V*) for SA at pH 5.0 at 25°C using PEDOT:PSS-PyBA electrodes. Mean±SD (*n*=5).


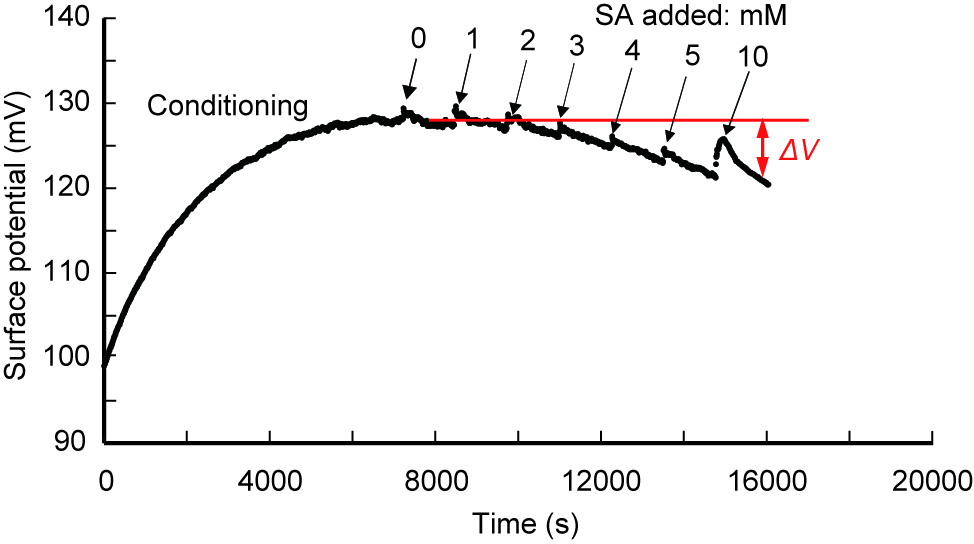


Figure S6. Time-course of potential for PEDOT:PSS-diamine during sequential increase of SA concentration from 0 to 10 mM at 20-min intervals in 10% FBS at pH 5.0 at 25̊C. *ΔV* was determined by the difference from the baseline just before treating the solution at 0 mM SA.
